# Obesity, metabolic risk and adherence to healthy lifestyle behaviours: prospective cohort study in the UK Biobank

**DOI:** 10.1186/s12916-022-02236-0

**Published:** 2022-02-15

**Authors:** Laura Heath, Susan A. Jebb, Paul Aveyard, Carmen Piernas

**Affiliations:** Nuffield Department of Primary Care Health Sciences, University of Oxford, Radcliffe Primary Care Building, Radcliffe Observatory Quarter, Woodstock Road, Oxford, OX2 6GG UK

**Keywords:** Obesity, Cardiovascular disease, Lifestyle factors, Metabolic complications of obesity, Weight management, Cohort study

## Abstract

**Background:**

Contested evidence suggests that obesity confers no risk to health in people who have a healthy lifestyle, particularly if there are no metabolic complications of obesity. The aim was to examine the association between adherence to lifestyle recommendations and the absence of metabolic complications on the incident or fatal cardiovascular disease and all-cause mortality across different categories of body mass index (BMI).

**Methods:**

This contemporary prospective cohort study included 339,902 adults without cardiovascular disease at baseline, recruited between 2006 and 2010 from the UK Biobank and followed until 2018–2020. The main exposures were four healthy lifestyle behaviours: never smoker, alcohol intake ≤ 112g/ week, 150 min moderate physical activity or 75 min vigorous activity/week, ≥ 5 servings of fruit or vegetables/day, and we assessed these overall and across the BMI groups. Metabolic complications of excess adiposity were hypertension, diabetes and hyperlipidaemia, and we examined whether obesity was associated with increased risk in the absence of these complications. The outcomes were all-cause mortality, death from, and incident cardiovascular disease (CVD).

**Results:**

Individuals who met four lifestyle recommendations but had excess weight had higher all-cause mortality; for BMI 30–34.9 kg/m^2^, the hazard ratio (HR) was 1.42 (95% confidence interval 1.20 to 1.68), and for BMI ≥ 35 kg/m^2^, HR was 2.17 (95% CI 1.71 to 2.76). The risk was lower, but still increased for people with no metabolic complications; for all-cause mortality, BMI 30–34.9 kg/m^2^ had an HR of 1.09 (95% CI 0.99 to 1.21), and BMI ≥ 35 kg/m^2^ had an HR of 1.44 (95% CI 1.19 to 1.74) for all-cause mortality. Similar patterns were found for incident and fatal CVD.

**Conclusions:**

Meeting healthy lifestyle recommendations, or the absence of metabolic complications of obesity offsets some, but not all, of the risk of subsequent CVD, and premature mortality in people with overweight or obesity. Offering support to achieve and maintain a healthy weight and to adopt healthy behaviours are likely to be important components in effective preventative healthcare.

**Supplementary Information:**

The online version contains supplementary material available at 10.1186/s12916-022-02236-0.

## Background

Adoption of healthy lifestyle behaviours has long been known to reduce the risk of CVD and death [1]. National and international policies and guidance from healthcare professionals have included smoking cessation, reduced alcohol intake, diets high in fruits and vegetables and increased physical activity as strategies to prevent ill health [2]. There is also a large body of evidence detailing the increased risks of excess adiposity, expressed as a raised BMI, on mortality [3]. This is of renewed concern as excess adiposity is strongly associated with an increased risk of hospitalisation and death from COVID-19 [4, 5].

Some studies, largely in the United States (US) health professional populations, have suggested that by adopting healthy behaviours, individuals with obesity could reduce their cardiovascular and all-cause mortality risk to equal to that of people with a BMI in the healthy range [6, 7]. This has led to the hypothesis that some people have a “fat but fit” phenotype, which posits that excess weight only confers additional risk if accompanied by low cardiorespiratory fitness [7]. Similarly, some authors have described a state of “metabolically healthy obesity” which is not necessarily associated with the adverse health outcomes usually associated with excess weight [8, 9]. More up-to-date evidence from general populations is needed to understand if excess weight is associated with additional risk, when healthy lifestyle recommendations are met and/or in the absence of metabolic complications. To date, policymakers have focussed on lifestyle factors, often targeted at those with metabolic complications of obesity. Understanding whether this is sufficient to overcome the adverse health implications associated with excess weight is essential to inform future obesity strategies.

Using a large contemporary UK cohort, we aimed to examine the association between adherence to four key lifestyle recommendations (never smoking, being physically active, consuming 5 or more portions of fruit and vegetables a day and moderate alcohol consumption) on all-cause mortality and fatal and total CVD risk overall and across BMI groups. Secondly, we examined the associations between the presence of metabolic complications (hypertension, diabetes, hyperlipidaemia) across the BMI groups with the same outcomes, as well as the interaction between the adherence to lifestyle behaviours and the presence of metabolic complications. To our knowledge, no study has examined both adherence to lifestyle behaviours, the presence of metabolic complications and the interaction between the two on all-cause mortality and fatal and total CVD across the BMI groups.

## Results

From the initial sample of 502,505 participants in the UK Biobank, participants were excluded prior to the analysis due to prior CVD (*n* = 37,108), BMI < 18.5 kg/m^2^ or missing (*n* = 5146), pregnancy (*n* = 368), missing data on smoking (*n* = 2211), alcohol (*n* = 101,397), fruits and vegetables (*n* = 5,852); and physical activity (*n* = 10,521) (Additional file 1: Fig. S1). Of the included population (*n* = 339,902), 52% were female and 96% white, and the mean BMI was 27.1 kg/m^2^ (standard deviation 4.4, Table 1). A comparison of baseline demographic factors between the included and excluded participants is recorded in the supplementary material (Additional file 2: Table. S1). Participants with missing data were a similar age, had a slightly higher baseline BMI and were more likely to be female than those without missing data. The mean follow-up period for mortality was 11.4 years (standard deviation 1.5 years), and incident CVD was 10.9 years (standard deviation 2.1 years). A total of 17,376 participants died during follow-up. Of these, 4653 were deaths from CVD, and 29,517 participants had a non-fatal CVD event.

**Table 1:** Participant characteristics at baseline according to the number of healthy behaviours

Factor	Total	Number of healthy behaviours*				
		0	1	2	3	4
*N*	339,902	30,101	93,063	118,254	77,287	21,197
Any death**	17,387 (5.1%)	2372 (7.9%)	5638 (6.1%)	5505 (4.7%)	3095 (4.0%)	777 (3.7%)
Incident cardiovascular disease (CVD)	29,545 (8.7%)	3506 (11.6%)	9105 (9.8%)	9837 (8.3%)	5709 (7.4%)	1388 (6.5%)
Male	161,849 (47.6%)	19,152 (63.6%)	53,760 (57.8%)	56,003 (47.4%)	27,446 (35.5%)	5488 (25.9%)
Age, mean (SD)	56.2 (8.0)	56.1 (7.9)	56.1 (8.0)	55.9 (8.1)	56.3 (8.1)	57.1 (7.9)
Body mass index (BMI), mean (SD)	27.1 (4.4)	27.8 (4.4)	27.4 (4.4)	27.0 (4.4)	26.7 (4.4)	26.5 (4.4)
Higher education	144,035 (42.4%)	12,302 (40.9%)	37,999 (40.8%)	50,269 (42.5%)	33,753 (43.7%)	9712 (45.8%)
White ethnicity	326,745 (96.1%)	29,405 (97.7%)	90,696 (97.5%)	113,940 (96.4%)	73,182 (94.7%)	19,522 (92.1%)
Current smoking	33,703 (9.9%)	8062 (26.8%)	15,468 (16.6%)	8555 (7.2%)	1618 (2.1%)	0 (0.0%)
Low fruit and vegetable intake	238,184 (70.1%)	30,101 (100.0%)	84,209 (90.5%)	85,564 (72.4%)	38,310 (49.6%)	0 (0.0%)
High alcohol consumption	188,151 (55.4%)	30,101 (100.0%)	78,082 (83.9%)	63,461 (53.7%)	16,507 (21.4%)	0 (0.0%)
Low physical activity	130,360 (38.4%)	30,101 (100.0%)	49,439 (53.1%)	40,673 (34.4%)	10,147 (13.1%)	0 (0.0%)
Hypertension	173,489 (51.0%)	16,973 (56.4%)	50,031 (53.8%)	58,864 (49.8%)	37,302 (48.3%)	10,319 (48.7%)
Diabetes	12,434 (3.7%)	1349 (4.5%)	3568 (3.8%)	4121 (3.5%)	2591 (3.4%)	805 (3.8%)
Hyperlipidaemia	138,069 (40.6%)	13,547 (45.0%)	39,367 (42.3%)	46,990 (39.7%)	29,849 (38.6%)	8316 (39.2%)
Post-menopause	106,899 (31.4%)	6245 (20.7%)	22,824 (24.5%)	36,706 (31.0%)	30,811 (39.9%)	10,313 (48.7%)
Family history of diabetes	60,259 (17.7%)	5030 (16.7%)	15,692 (16.9%)	21,033 (17.8%)	14,365 (18.6%)	4139 (19.5%)
Family history of CVD	200,113 (58.9%)	17,148 (57.0%)	54,007 (58.0%)	69,870 (59.1%)	46,350 (60.0%)	12,738 (60.1%)

*Healthy behaviours defined as the total of being a never smoker, consuming ≥ 5 portions of fruit/vegetables a day, meeting international physical activity guidance, and drinking ≤ 112 g alcohol a week

**Figures are *n* (%) unless otherwise stated

**Fig. 1:**
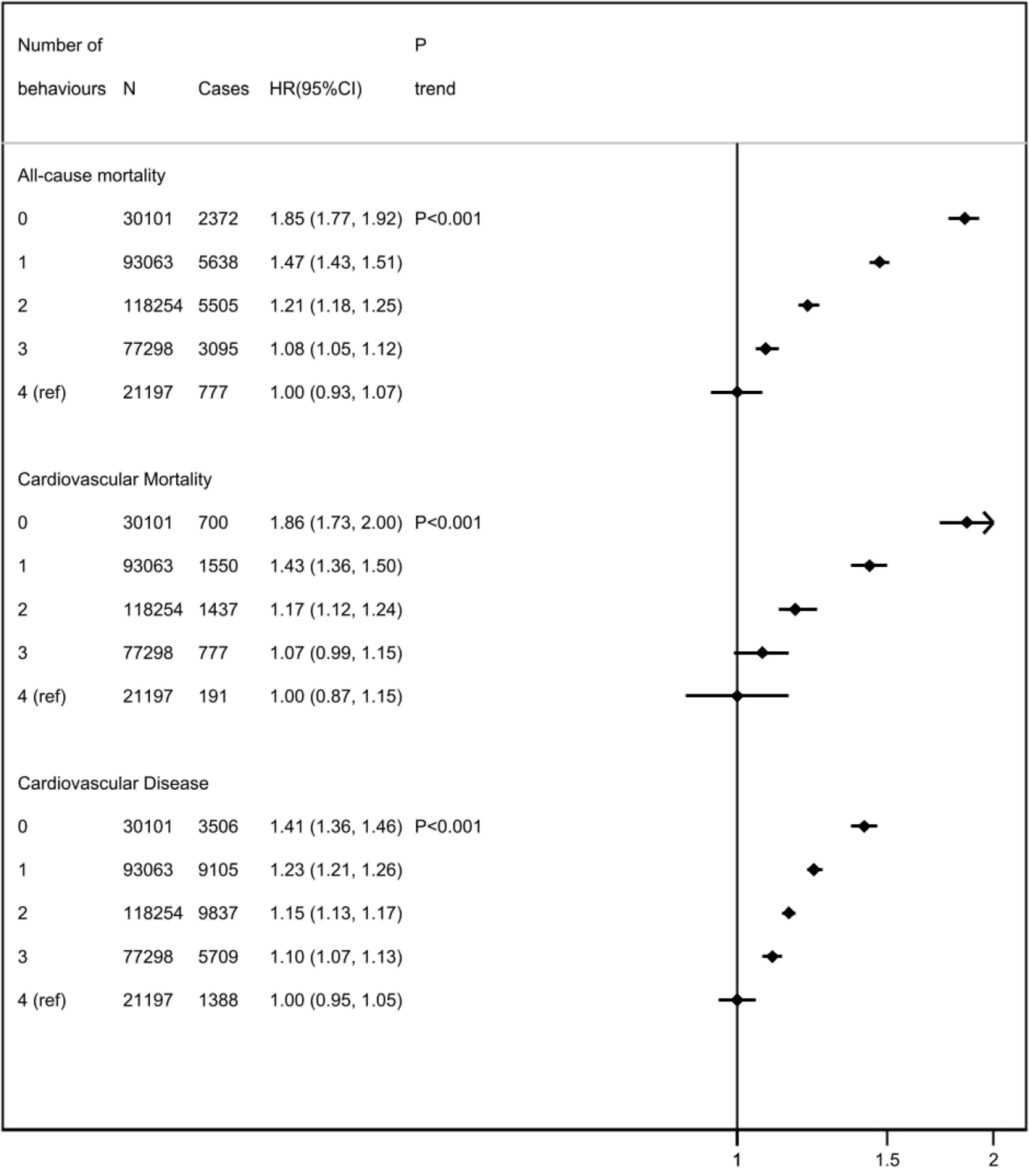
Cox proportional hazards models for risk of all-cause mortality, cardiovascular mortality, or incident cardiovascular disease by adherence to 0, 1, 2, 3 or 4 healthy lifestyle behaviours. HR, hazard ratio; CI, confidence interval (floating). Multivariable hazard ratio adjusted for age, sex, ethnicity, Townsend Deprivation Score, education, region, family history of cardiovascular disease, family history of diabetes, menopausal status, and BMI group

**Fig. 2:**
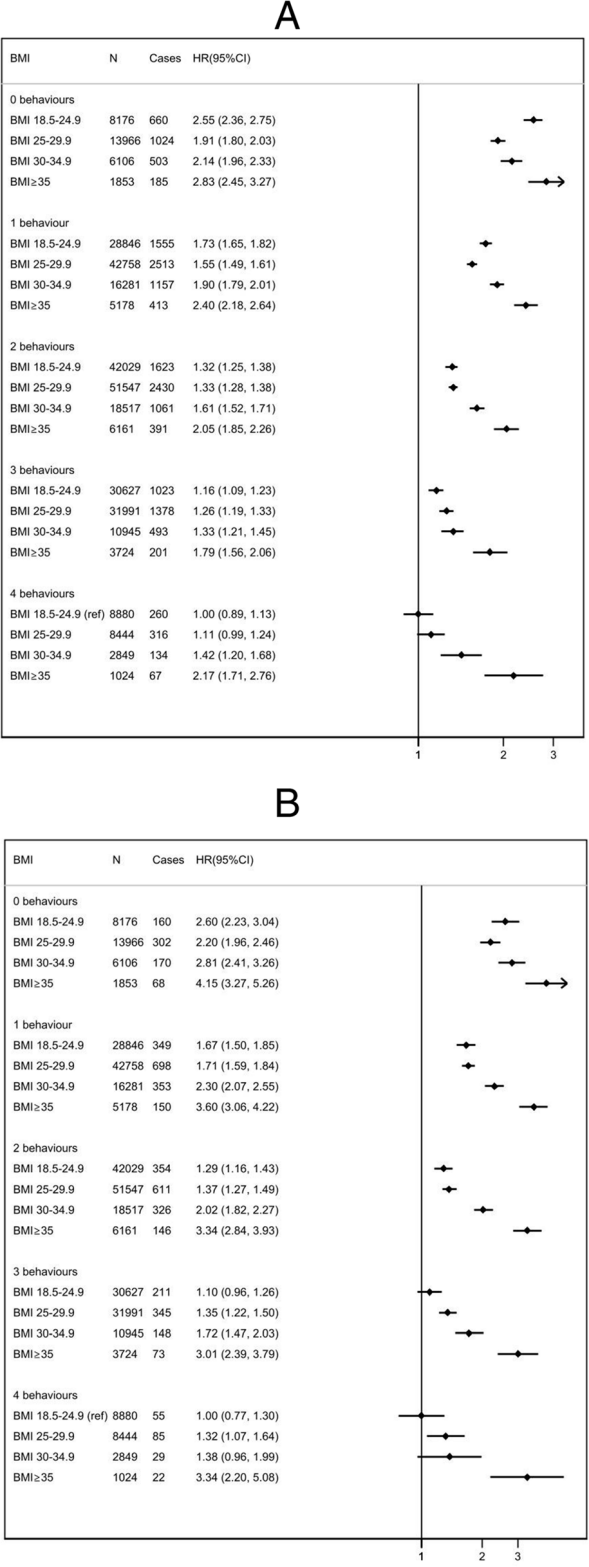

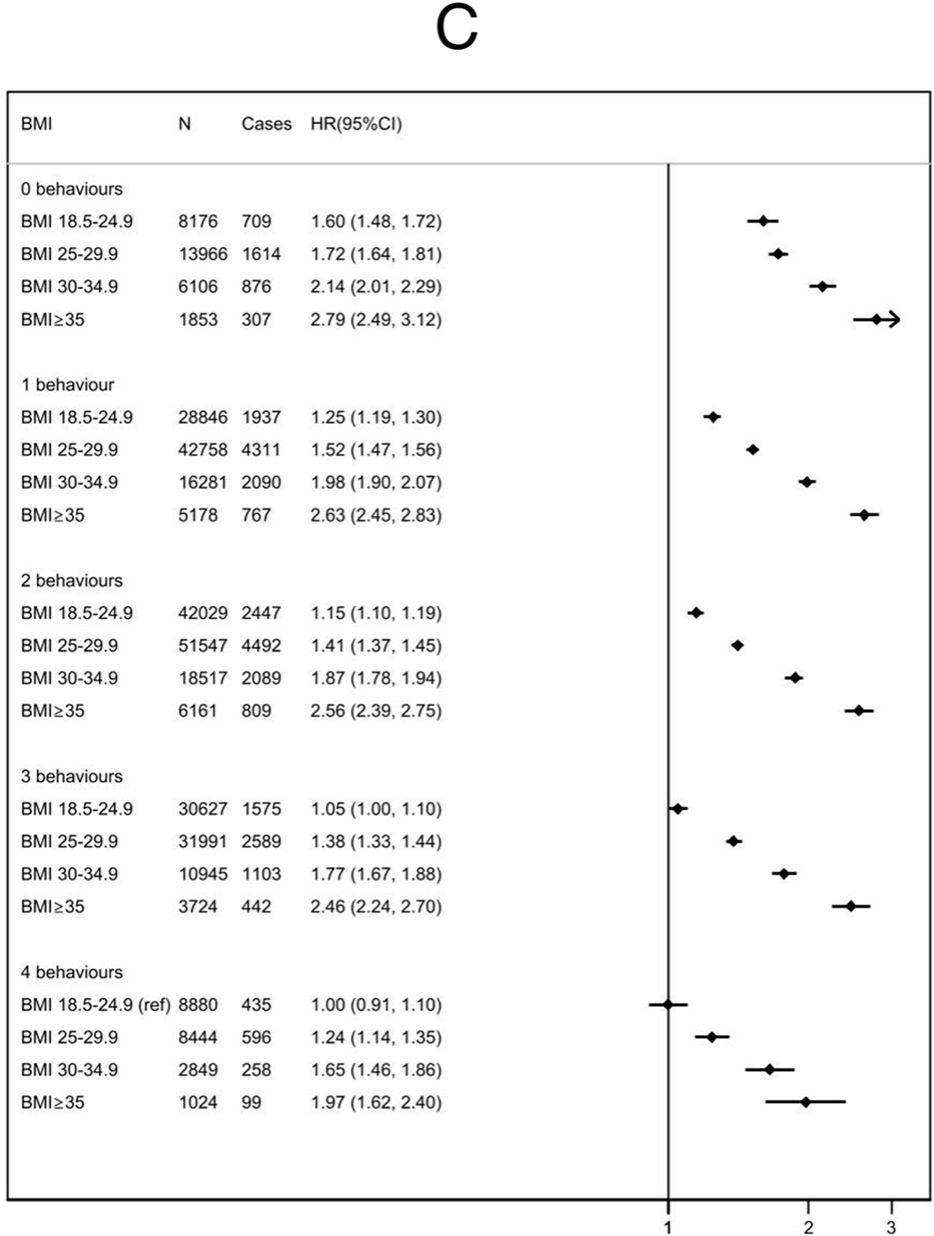
Cox proportional hazards models for risk of all-cause mortality (**A**), cardiovascular mortality (**B**), and incident cardiovascular disease (**C**) by BMI group (kg/m^2^), stratified by adherence to 0, 1, 2, 3, or 4 healthy lifestyle behaviours. HR, hazard ratio; CI, confidence interval (floating); BMI, body mass index (kg/m^2^). Multivariable hazard ratio adjusted for age, sex, ethnicity, Townsend Deprivation Score, education, region, family history of cardiovascular disease, family history of diabetes, and menopausal status

There was an association between meeting an increasing number of healthy lifestyle recommendations and a reduced incidence of CVD, fatal CVD or all-cause mortality (Fig. 1). The association was similar when stratified by BMI category, although the relative reduction in risk associated with the number of protective behaviours was smaller for people with higher BMI (Fig. 2). Amongst the people meeting all four healthy lifestyle behaviours who were overweight, the hazard ratio (HR) was 1.11 (95% confidence interval (CI) 0.99 to 1.24); for people with obesity, it was 1.42 (95% CI 1.20 to 1.68); and those with severe obesity, it was 2.17 (95% CI 1.71 to 2.76). Similar patterns were observed with CVD mortality and incidence (Fig. 2). There was evidence of increased risk of all-cause mortality (*p*_trend_ < 0.001), cardiovascular mortality (*p*_trend_ < 0.001) and cardiovascular disease (*p*_trend_ < 0.001) as the BMI group increased.

In each BMI subgroup, meeting each individual lifestyle recommendation was associated with a lower risk of all-cause mortality and CVD incidence than not meeting it. The associations with smoking were very strong and only modest for the other lifestyle risk factors (Table 2).

**Table 2:** Cox proportional hazards models for risk of all-cause mortality, cardiovascular mortality, or cardiovascular disease diagnosis by adherence to individual healthy behaviour, stratified by BMI group

	All-cause mortality			Cardiovascular mortality				Incident cardiovascular disease				
BMI, kg/m^2^	18.5–24.9	25–29.9	30–34.9	≥ 35	18.5–24.9	25–29.9	30–34.9	≥ 35	18.5–24.9	25–29.9	30–34.9	≥ 35
*Smoking*												
Never (reference)	1.00	1.00	1.00	1.00	1.00	1.00	1.00	1.00	1.00	1.00	1.00	1.00
Previous	1.25 (1.18–1.34)	1.25 (1.19–1.32)	1.27 (1.18–1.37)	1.27 (1.13–1.43)	1.25 (1.09–1.43)	1.25 (1.14–1.38)	1.19 (1.04–1.37)	1.13 (0.92–1.37)	1.12 (1.07–1.18)	1.13 (1.09–1.17)	1.14 (1.08–1.20)	1.16 (1.07–1.26)
Current	3.07 (2.86–3.30)	2.29 (2.14–2.44)	2.01 (1.80–2.23)	1.85 (1.54–2.22)	3.49 (3.01–4.05)	2.71 (2.40–3.07)	2.29 (1.90–2.75)	1.89 (1.41–2.54)	1.96 (1.84–2.10)	1.69 (1.60–1.78)	1.54 (1.42–1.67)	1.59 (1.39–1.82)
*Alcohol (g/week)*												
None	1.27 (1.08–1.50)	1.00 (0.85–1.17)	1.21 (1.00–1.47)	1.12 (0.87–1.44)	1.52 (1.10–2.11)	1.00 (0.73–1.37)	1.07 (0.72–1.57)	1.06 (0.68–1.65)	1.18 (1.03–1.37)	1.10 (0.98–1.23)	1.37 (1.20–1.57)	1.21 (1.01–1.44)
Occasional (< 8)	1.32 (1.09–1.61)	1.11 (0.93–1.31)	1.08 (0.85–1.38)	1.22 (0.91–1.62)	1.32 (0.87–2.01)	1.11 (0.78–1.57)	1.14 (0.72–1.79)	1.61 (1.02–2.51)	1.10 (0.93–1.31)	1.10 (0.97–1.25)	1.14 (0.97–1.35)	1.37 (1.12–1.66)
Moderate (8–112) reference	1.00	1.00	1.00	1.00	1.00	1.00	1.00	1.00	1.00	1.00	1.00	1.00
Heavy I (112–168)	0.97 (0.89–1.05)	0.89 (0.83–0.96)	1.08 (0.97–1.20)	1.09 (0.91–1.29)	0.81 (0.67–0.98)	0.88 (0.77–1.01)	1.07 (0.88–1.31)	1.04 (0.77–1.40)	0.94 (0.88–1.01)	0.98 (0.93–1.03)	0.94 (0.87–1.01)	0.96 (0.84–1.09)
Heavy II (168–224)	0.95 (0.86–1.04)	0.95 (0.88–1.02)	0.88 (0.78–0.99)	1.18 (0.98–1.43)	0.80 (0.65–0.99)	0.87 (0.75–1.01)	0.83 (0.66–1.05)	1.28 (0.94–1.74)	0.94 (0.87–1.02)	0.94 (0.89–1.00)	0.99 (0.91–1.07)	1.10 (0.95–1.25)
Heavy III (> 224)	1.23 (1.15–1.32)	1.00 (0.95–1.06)	1.12 (1.04–1.22)	1.09 (0.95–1.25)	1.30 (1.13–1.50)	1.05 (0.94–1.16)	1.21 (1.04–1.41)	1.04 (0.82–1.31)	1.05 (0.99–1.12)	0.96 (0.92–1.01)	1.02 (0.96–1.08)	1.04 (0.94–1.15)
*Fruit/vegetables (servings/day)*												
≥ 5 (reference)	1.00	1.00	1.00	1.00	1.00	1.00	1.00	1.00	1.00	1.00	1.00	1.00
< 5	1.17 (1.10–1.25)	1.03 (0.98–1.08)	1.02 (0.94–1.10)	0.98 (0.87–1.10)	1.12 (0.98–1.28)	1.07 (0.97–1.18)	1.02 (0.89–1.17)	1.08 (0.89–1.32)	1.08 (1.03–1.14)	1.05 (1.01–1.09)	1.03 (0.97–1.08)	0.97 (0.89–1.06)
*Meeting physical activity guidelines*												
Yes (reference)	1.00	1.00	1.00	1.00	1.00	1.00	1.00	1.00	1.00	1.00	1.00	1.00
No	1.20 (1.14–1.27)	1.09 (1.04–1.15)	1.13 (1.06–1.21)	1.08 (0.96–1.20)	1.25 (1.11–1.41)	1.06 (0.97–1.16)	1.17 (1.03–1.32)	0.95 (0.79–1.15)	1.07 (1.02–1.13)	1.03 (0.99–1.07)	1.05 (1.00–1.10)	1.05 (0.97–1.14)

Values presented as HR (95% CI); multivariable hazard ratio adjusted for age, sex, ethnicity, Townsend Deprivation Score, education, region, family history of cardiovascular disease, family history of diabetes, and menopausal status

**Fig. 3:**
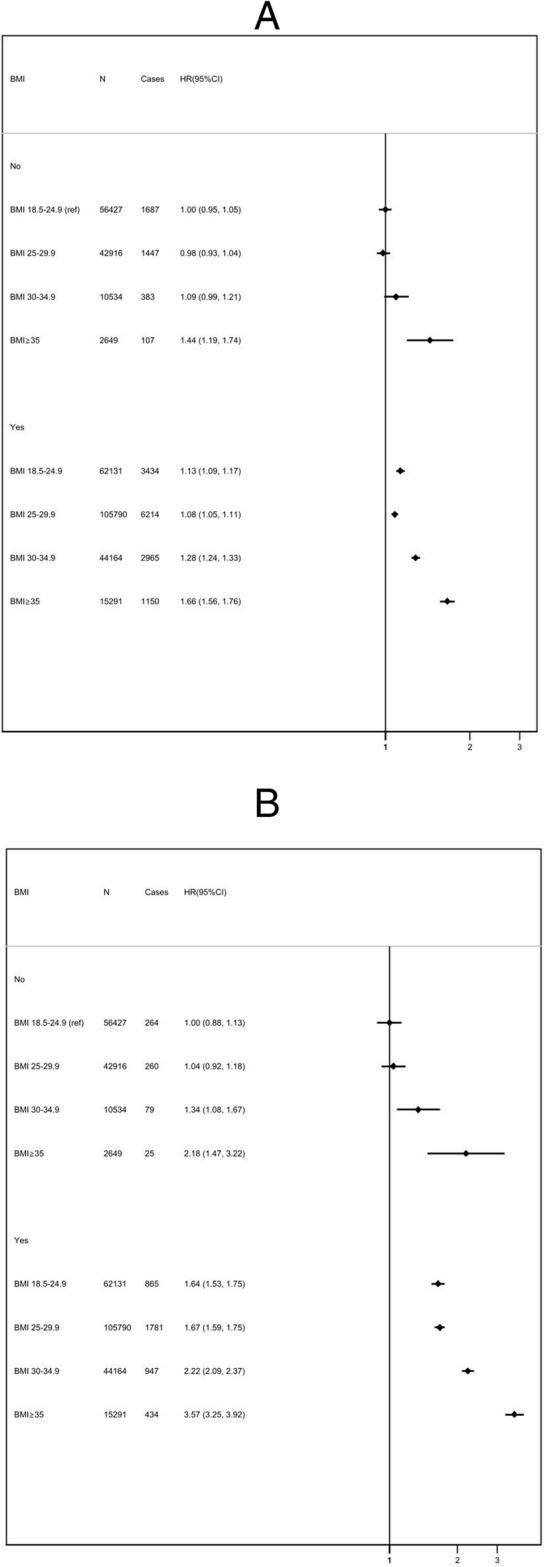

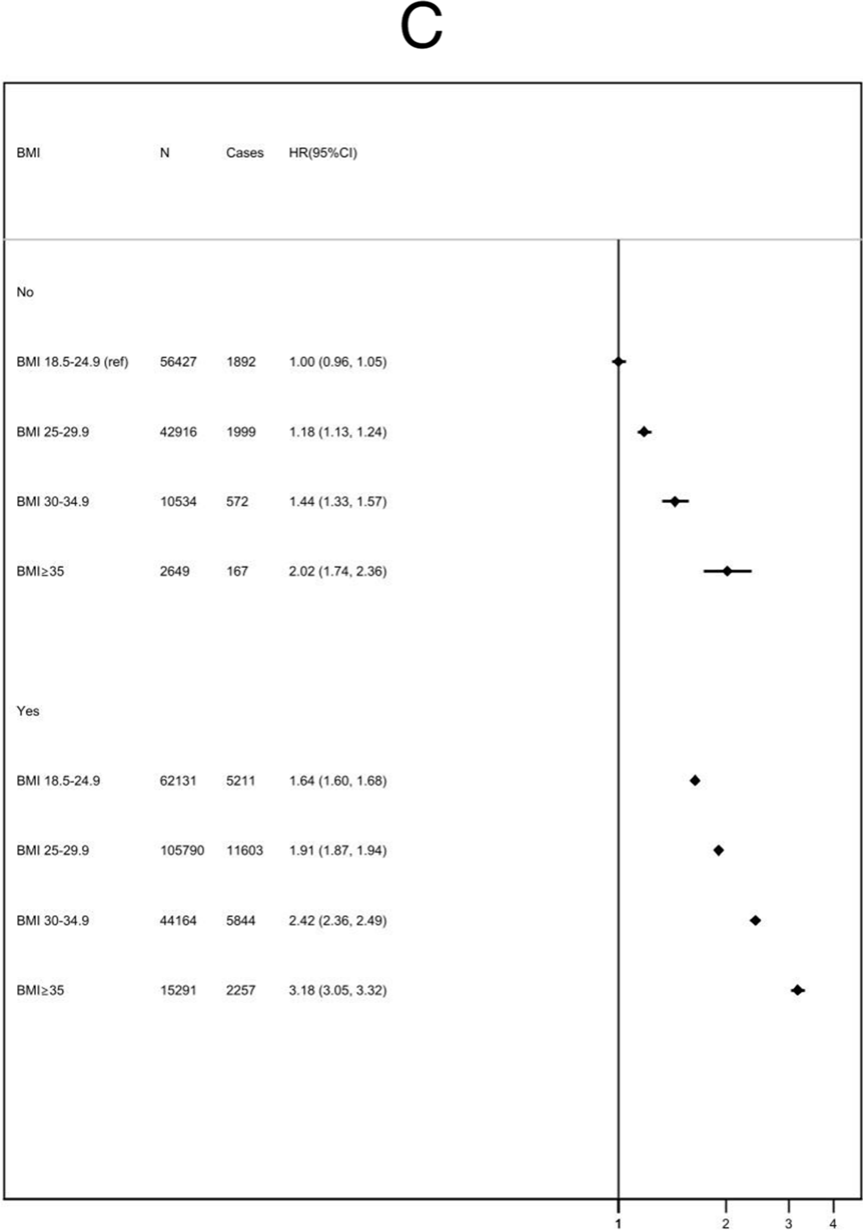
Cox proportional hazards models for risk of all-cause mortality (**A**), cardiovascular mortality (**B**), and incident cardiovascular disease (**C**) by BMI group (kg/m^2^), stratified according to the presence or absence of metabolic risk factors. HR, hazard ratio; CI, confidence intervals (floating); BMI, body mass index (kg/m^2^). Metabolically “healthy” includes patients without a diagnosis of hypertension, diabetes, or hyperlipidaemia. Multivariable hazard ratio adjusted for age, sex, ethnicity, Townsend Deprivation Score, education, region, family history of cardiovascular disease, family history of diabetes, and menopausal status

The presence of metabolic complications was associated with an increased risk of all adverse outcomes in every BMI subgroup (Figure 3). The relative increase in risk associated with metabolic complications was greater for higher BMI groups. For people who were overweight with no metabolic complications, the HR was 0.98 (95% CI 0.93 to 1.04); for people with obesity, it was 1.09 (95% CI 0.99 to 1.21); and those with severe obesity, it was 1.44 (95% CI 1.19 to 1.724). There was no evidence that the presence of metabolic complications modified the relationship between meeting lifestyle recommendations and incidence of CVD, fatal CVD or all-cause mortality across BMI strata (*p* = 0.77).

Sensitivity analysis excluding the first 2 years of follow-up showed consistent results (Additional file 3: Table. S1- Table. S3).

## Discussion

In this contemporary cohort of the UK general population, adherence to healthy lifestyle recommendations was sufficient to offset some, but not all, all-cause mortality risk associated with obesity (BMI ≥ 30 kg/m^2^) compared to those of a healthy BMI who met all four healthy lifestyle recommendations. At more extreme levels of obesity (BMI ≥ 35 kg/m^2^), those who adhered to all four healthy lifestyle behaviours, although substantially reducing their risk, still had a 2–3-fold increased risk of death compared to those of a healthy BMI who met all four healthy lifestyle recommendations. The trend was similar for cardiovascular mortality and incident cardiovascular disease.

The findings amongst people classified as overweight (BMI 25–30 kg/m^2^) were mixed. There was consistent evidence of increased incident CVD, but not cardiovascular or all-cause mortality amongst participants who met lifestyle recommendations or who had no underlying cardiometabolic complications. We suspect the protective effect of overweight in older adults or the impact of medical interventions to manage risk factors may explain why there was an increased risk of incident CVD, but not mortality in this BMI group, as described in previous studies [10].

This analysis reinforces the message that promoting healthy behaviours is additional to, and not a substitute for, interventions to support people to achieve and maintain a healthy weight, regardless of metabolic complications. This should encourage clinicians to make greater use of the interventions to support weight loss as a key component of preventative healthcare. Advice and support to encourage people to meet healthy lifestyle recommendations will bring additional health benefits, independent of weight. However, governments and policymakers should be aware that advocating for lifestyle change alone is insufficient to overcome some of the health risks associated with excess weight.

Of note, individuals with a healthy BMI who did not meet any healthy lifestyle recommendations were amongst those most at risk of death in this cohort. Previous analyses have found that a substantial proportion of leanness in the USA and Europe is driven by exposure to smoking, undernutrition and alcohol intake, and our results support this conclusion [11]. There may also be some reverse causality since some people with incipient chronic disease may not have been excluded at baseline, e.g. undiagnosed cancer. Nonetheless, this finding is important for clinicians, as discussions about a healthy diet, smoking cessation or the importance of physical activity may only be initiated for those patients with excess weight or metabolic complications [12]. Yet those with a BMI in the healthy range may also derive significant benefit from these interventions. Given that adherence to lifestyle recommendations is low, there is also a need for population-level policies to support people to adopt these healthier behaviours.

A strength of this study is the sample size in higher BMI subgroups, especially in the group of people meeting four recommendations, or with no cardiometabolic complications, which is favourable compared to existing studies on this subject [6]. Another strength is the end points (death), and diagnosis of cardiovascular disease were based on reliable death registries and universal recording of hospital admissions with ICD-10 coding for cardiovascular diseases. A wide range of covariates was also available to adjust for confounding, although residual confounding cannot be excluded, as in any observational analysis. Similarly, we cannot rule out reverse causality (for example, reduced physical activity caused by incipient cardiovascular disease), although our sensitivity analysis excluding participants with a diagnosis of cardiovascular disease within 2 years of follow-up showed a similar pattern of results.

A limitation is that only 5.5% of those invited enrolled in the UK Biobank, and therefore, the study population may not be completely representative of the UK population in terms of lifestyle. Participants are less likely to smoke, to drink alcohol regularly or to have raised BMI than the general population. However, a recent study found similar associations between the risk factors and mortality end points in the UK Biobank cohort compared to other more representative nationwide surveys, which provides reassurance that the associations reported here are likely to be robust [13]. Participants excluded from this study due to missing data in key exposures (i.e. alcohol intake) were found to be slightly different than those included (as shown in Appendix Table 1). Multiple imputation was not considered here as data may be missing not at random (MNAR) [14]. Although our analysis adjusted for all major confounders, we were not able to adjust for current medications (i.e., those prescribed after the baseline appointment), which may reduce the development of CVD and mortality. Physical activity, fruit and vegetables and smoking data were self-reported, but in this prospective evaluation, this error is unlikely to be related to the outcomes of interest and therefore less prone to influence the associations. Other studies chose to subdivide healthy BMI into two categories 18.5–22.4 kg/m^2^ and 22.5–25 kg/m^2^ [11]. This is helpful to investigate the U-shaped association between BMI and mortality. However, here, our focus was on the risks associated with excess adiposity, and we therefore subdivided all obesity (BMI ≥ 30 kg/m^2^) into obesity (BMI 30–34.9 kg/m^2^) and severe obesity (BMI ≥ 35 kg/m^2^). In addition, the UK Biobank collected data on lifestyle behaviour for the entire sample only at baseline, which does not account for possible changes in behaviours over time. For a more comprehensive view of exposure, future cohort studies should consider measuring behavioural factors at multiple time points to account for the changes that may affect the outcomes.

This analysis is supported by a recent large cohort study that also found that people with BMI 18.5–22.4 kg/m^2^ who adhered to all healthy lifestyle recommendations have the lowest risk of premature mortality [11]. Similarly, another cohort study found adherence to a healthy Mediterranean diet can offset some, but not all, of the excess CVD mortality associated with obesity [15]. The present analysis adds to this work by further considering severe obesity and whether the presence of metabolic complications modifies the relationship between meeting lifestyle recommendations, and cardiovascular risk or all-cause mortality. Unlike a US cohort study that found no evidence of an increase in all-cause mortality in people with obesity who adhered to all lifestyle recommendations, this analysis shows the clear residual risk for people with excess weight [6].

Unsurprisingly, and in common with a recent UK Biobank analysis, we found a reduced cardiovascular risk for people with obesity and no metabolic complications. However, the risk for these people was still higher than their peers with no metabolic complications, but who also had a healthy BMI [16]. The present study additionally found no evidence that this difference was modified by adherence to healthy lifestyle behaviours.

Other prospective cohort studies found metabolically healthy obesity was associated with increased risk of developing diabetes [17, 18], ischaemic heart disease [19], CVD [20], all-cause mortality [21], poorer quality of life scores [22] and may be a transitory state [23]. Recent systematic reviews on metabolically healthy obesity and cardiovascular risk support these findings [24–27]. In general, the cohort studies that have reported that there was no increase in CVD or all-cause mortality have had smaller sample sizes and shorter follow-up and may have been underpowered to detect associations with adverse outcomes [28]. Excess weight is also known to confer other risks not assessed here, such as musculoskeletal problems, which are responsible for considerable morbidity, and these are mechanistically unlikely to be offset by healthy lifestyle behaviours [29].

## Conclusions

In conclusion, this analysis shows that adopting healthy lifestyle recommendations is of significant benefit to all, including those with a healthy weight. However, it is insufficient to offset all of the cardiovascular risk and premature mortality associated with excess weight, irrespective of whether there was evidence of metabolic complications of obesity. Policies that support the whole population to achieve and maintain a healthy weight even for people with obesity who meet healthy lifestyle recommendations or those with no metabolic complications are likely to be beneficial.

## Methods

### Design and study population

The UK Biobank study is a national prospective cohort that recruited 502,505 participants aged 40–69 years between 2006 and 2010 [30]. A wide range of information on socio-demographic characteristics and behavioural factors was collected, along with physical measurements (such as height and weight), blood and urine samples. UK Biobank protocols and study details can be found elsewhere [30]. The UK Biobank study was conducted according to the Declaration of Helsinki, and ethical approval was granted by the North West Multi-Centre Research Ethics Committee (reference number 06/MRE08/65). At recruitment, all participants gave informed consent to participate and be followed up through data linkage.

### Exclusions

We excluded participants who had a BMI < 18.5 kg/m^2^; who were pregnant; who had a diagnosis of CVD, including coronary heart disease, congestive heart failure, cardiomyopathy or stroke at baseline; or who did not have information on the four healthy lifestyle behaviours detailed in the exposures, or key covariates at baseline.

### Exposures

Adherence to four lifestyle recommendations was determined using data from the self-administered questionnaires at baseline. The four healthy lifestyle exposures were defined as follows: [1] “never” smoker, 0 cigarettes/day throughout life [2]; alcohol intake frequency, < 112 g/week in line with the UK guidance on low alcohol intake [31] [3]; 5 or more servings of fruit and vegetable/day in line with the World Health Organization (WHO) guidance on healthy diet [32]. Fruit and vegetable intake was selected as the healthy diet indicator, as a dietary risk factor in its own right and as a proxy for a healthy diet [33] [4]; 150 min/week or more of moderate-vigorous activity a week as defined by the WHO physical activity guidelines [34].

Cardiometabolic health was determined by the presence or absence of metabolic complications, specifically hypertension, diabetes or hyperlipidaemia at baseline. Hypertension was defined as either a medical diagnosis of hypertension, taking medication for hypertension, having an average systolic blood pressure ≥ 140 mmHg or having an average diastolic blood pressure ≥ 90 mmHg. Diabetes was defined as having a medical diagnosis of diabetes or taking medication for diabetes. Hyperlipidaemia was defined as taking cholesterol-lowering medication or low-density lipoprotein (LDL) > 4 mmol/L. These three markers of cardiometabolic health are known to mediate some of the adverse health outcomes of obesity [35] and to aid comparability with other studies [20].

BMI was categorised as follows: BMI 18.5–24.9 kg/m^2^ “normal”, 25–29.9 kg/m^2^ “overweight”, 30–34.9 kg/m^2^ “obesity” and ≥ 35 kg/m^2^ “severe obesity”. The category 18.5–24.9 kg/m^2^ was the reference group [36].

### Outcomes

The main outcomes were incidence of CVD, death from CVD and all-cause mortality. Incident cardiovascular disease was defined as a hospital admission or death with the International Classification of Diseases, 10th revision (ICD-10) codes including coronary heart disease (CHD; I20–I25), congestive heart failure or cardiomyopathy (CHF; I50, I50.1, 150.9, I11.0, I13.0, I13.2, I42, I43.1) and total stroke (I60–I64). Hospital admission data were available until September 30, 2020, in England; August 31, 2020, in Scotland; and February 28, 2018, in Wales. Death registries included the date of deaths if occurred before September 30, 2020, in England, Wales and Scotland.

### Statistical analysis

The analysis followed a pre-specified statistical plan (version 3.0 dated October 21, 2020) (Additional file 4). The primary analysis aimed to investigate the association between the number of lifestyle recommendations met and incident CVD, fatal CVD and total mortality overall, and within each BMI strata. We also investigated the associations of each individual behaviour with incident CVD, fatal CVD and total mortality across the four BMI strata. For all analyses, the number of recommendations met (0, 1, 2, 3 or 4) out of a total of 4 were used as the main exposure, using 4 as the reference category.

A secondary analysis was performed to examine the associations between the presence of metabolic complications (hypertension, diabetes, hyperlipidaemia) across the BMI groups following the approach described above. Additionally, a triple interaction between adherence to lifestyle behaviours, presence of metabolic complications and BMI group was tested with a likelihood ratio test to compare a model with vs without interaction. Metabolic health was a binary variable, defined by the presence or absence of metabolic complications (hypertension, diabetes or hyperlipidaemia) with the absence of metabolic complications as the reference category.

Multivariable Cox proportional hazards models were used to estimate hazard ratios (HRs) with 95% CIs, using floated absolute risks [37]. The proportional hazards assumption was based on Schoenfeld residuals and was not violated for the variables of interest in the adjusted model (*p* > 0.05). To calculate the time to follow-up, we used age at completion of the baseline questionnaires as the start date until the age of occurrence of the first event (CVD or death) or censoring date, whichever came first. Analyses were stratified by sex (male or female) and UK region (but pooled into a single estimate) and adjusted for age (underlying time co-variate in the Cox model), Townsend Deprivation Score (quintiles 1–5, with a lower score representing greater affluence), education (1: higher degree, 2: any school degree, 3: vocational qualifications, 0: none of the above), family history of diabetes (yes or no), family history of CVD (yes or no) and menopausal status (age ≥ 55 years used as a proxy if menopausal status was missing). Sensitivity analysis excluded participants who had the event within the first 2 years of follow-up, to rule out the probability of reversed causality. A post hoc trend analysis tested whether there was evidence of increased risk of adverse health outcomes for different BMI groups across the number of healthy recommendations met. All analyses were conducted in Stata (version 14.2).

## Supplementary Information

**Additional file 1: Figure S1.** Flow chart through the study.

**Additional file 2: Table S1**. Complete Data vs Missing Data Participant Characteristics at Baseline.

**Additional file 3: Table S1.** Cox Proportional Hazards Models for Risk of All-Cause Mortality, Cardiovascular Mortality, or Cardiovascular Disease Diagnosis by Adherence to 0, 1, 2, 3, or 4 Healthy Lifestyle Behaviours Excluding First 2 Years of Follow Up. **Table S2.** Cox Proportional Hazards Models for Risk of All-Cause Mortality, Cardiovascular Mortality, or Cardiovascular Disease Diagnosis by Adherence to 0, 1, 2, 3, or 4 Healthy Lifestyle Behaviours, Stratified by BMI Group, Excluding First 2 Years of Follow Up. **Table S3.** Cox Proportional Hazards Models for Risk of All-Cause Mortality, Cardiovascular Mortality, or Cardiovascular Disease Diagnosis by According to Presence or Absence of Metabolic Risk Factors, Stratified by BMI Group, Excluding First 2 Years of Follow Up.

**Additional file 4:.** Statistical Analysis Plan: Healthy behaviours, cardiovascular disease and all-cause mortality in the UK Biobank.

## Data Availability

UK Biobank data is available to researchers on application (https://www.ukbiobank.ac.uk/enable-your-research).

## References

[1] Global Burden of Disease Risk Factor Collaborators (2018). Global, regional, and national comparative risk assessment of 84 behavioural, environmental and occupational, and metabolic risks or clusters of risks for 195 countries and territories, 1990-2017: a systematic analysis for the Global Burden of Disease Study 2017. Lancet.

[2] World Health Organization. Disease prevention 2020 [Available from: https://www.euro.who.int/en/health-topics/disease-prevention.

[3] Kivimaki M, Kuosma E, Ferrie JE, Luukkonen R, Nyberg ST, Alfredsson L (2017). Overweight, obesity, and risk of cardiometabolic multimorbidity: pooled analysis of individual-level data for 120 813 adults from 16 cohort studies from the USA and Europe. Lancet Public Health..

[4] Hussain A, Mahawar K, Xia Z, Yang W, El-Hasani S (2020). Obesity and mortality of COVID-19. Meta-analysis. Obes Res Clin Pract..

[5] Yang J, Hu J, Zhu C (2020). Obesity aggravates COVID-19: a systematic review and meta-analysis. J Med Virol..

[6] Matheson EM, King DE, Everett CJ (2012). Healthy lifestyle habits and mortality in overweight and obese individuals. JABFM.

[7] Barry VW, Baruth M, Beets MW, Durstine JL, Liu J, Blair SN (2014). Fitness vs. fatness on all-cause mortality: a meta-analysis. Prog Cardiovasc Dis..

[8] Stefan N, Haring HU, Hu FB, Schulze MB (2013). Metabolically healthy obesity: epidemiology, mechanisms, and clinical implications. Lancet Diabetes Endocrinol..

[9] Iacobini C, Pugliese G, Blasetti Fantauzzi C, Federici M, Menini S (2019). Metabolically healthy versus metabolically unhealthy obesity. Metabolism..

[10] Winter JE, MacInnis RJ, Wattanapenpaiboon N, Nowson CA (2014). BMI and all-cause mortality in older adults: a meta-analysis. Am J Clin Nutr..

[11] Veronese N, Li Y, Manson JE, Willett WC, Fontana L, Hu FB (2016). Combined associations of body weight and lifestyle factors with all cause and cause specific mortality in men and women: prospective cohort study. BMJ..

[12] Gupta R, Wood DA (2019). Primary prevention of ischaemic heart disease: populations, individuals, and health professionals. Lancet..

[13] Batty GD, Gale CR, Kivimaki M, Deary IJ, Bell S (2020). Comparison of risk factor associations in UK Biobank against representative, general population based studies with conventional response rates: prospective cohort study and individual participant meta-analysis. BMJ..

[14] Jakobsen JC, Gluud C, Wetterslev J, Winkel P (2017). When and how should multiple imputation be used for handling missing data in randomised clinical trials - a practical guide with flowcharts. BMC Med Res Methodol..

[15] Michaelsson K, Baron JA, Byberg L, Hoijer J, Larsson SC, Svennblad B (2020). Combined associations of body mass index and adherence to a Mediterranean-like diet with all-cause and cardiovascular mortality: a cohort study. PLoS Med..

[16] Zhou Z, Macpherson J, Gray SR, Gill JMR, Welsh P, Celis-Morales C, Sattar N, Pell JP, Ho FK (2021). Are people with metabolically healthy obesity really healthy? A prospective cohort study of 381,363 UK Biobank participants. Diabetologia..

[17] Hsu ARC, Ames SL, Xie B, Peterson DV, Garcia L, Going SB (2020). Incidence of diabetes according to metabolically healthy or unhealthy normal weight or overweight/obesity in postmenopausal women: the Women’s Health Initiative. Menopause..

[18] Wei Y, Wang J, Han X, Yu C, Wang F, Yuan J, Miao X, Yao P, Wei S, Wang Y, Liang Y, Zhang X, Guo H, Zheng D, Tang Y, Yang H, He M (2020). Metabolically healthy obesity increased diabetes incidence in a middle-aged and elderly Chinese population. Diabetes Metab Res Rev..

[19] Hansen L, Netterstrom MK, Johansen NB, Ronn PF, Vistisen D, Husemoen LLN (2017). Metabolically healthy obesity and ischemic heart disease: a 10-year follow-up of the Inter99 Study. J Clin Endocrinol Metab..

[20] Caleyachetty R, Thomas GN, Toulis KA, Mohammed N, Gokhale KM, Balachandran K, Nirantharakumar K (2017). Metabolically healthy obese and incident cardiovascular disease events among 3.5 million men and women. J Am Coll Cardiol..

[21] Kuk JL, Ardern CI (2009). Are metabolically normal but obese individuals at lower risk for all-cause mortality?. Diabetes Care..

[22] Lopez-Garcia E, Guallar-Castillon P, Garcia-Esquinas E, Rodriguez-Artalejo F (2017). Metabolically healthy obesity and health-related quality of life: a prospective cohort study. Clin Nutr..

[23] Appleton SL, Seaborn CJ, Visvanathan R, Hill CL, Gill TK, Taylor AW, Adams RJ, on behalf of the North West Adelaide Health Study Team (2013). Diabetes and cardiovascular disease outcomes in the metabolically healthy obese phenotype: a cohort study. Diabetes Care..

[24] Eckel N, Meidtner K, Kalle-Uhlmann T, Stefan N, Schulze MB (2016). Metabolically healthy obesity and cardiovascular events: a systematic review and meta-analysis. Eur J Prev Cardiol..

[25] Opio J, Croker E, Odongo GS, Attia J, Wynne K, McEvoy M (2020). Metabolically healthy overweight/obesity are associated with increased risk of cardiovascular disease in adults, even in the absence of metabolic risk factors: a systematic review and meta-analysis of prospective cohort studies. Obes Rev..

[26] Yeh TL, Chen HH, Tsai SY, Lin CY, Liu SJ, Chien KL. The relationship between metabolically healthy obesity and the risk of cardiovascular disease: a systematic review and meta-analysis. J Clin Med. 2019;8(8):1228. 10.3390/jcm8081228 PMC6723711 31443279

[27] Kramer CK, Zinman B, Retnakaran R (2013). Are metabolically healthy overweight and obesity benign conditions?: a systematic review and meta-analysis. Ann Intern Med..

[28] Hamer M, Stamatakis E (2012). Metabolically healthy obesity and risk of all-cause and cardiovascular disease mortality. J Clin Endocrinol Metab..

[29] O’Halloran R, Mihaylova B, Cairns BJ, Kent S (2020). BMI and cause-specific hospital admissions and costs: the UK Biobank Cohort Study. Obesity (Silver Spring)..

[30] Sudlow C, Gallacher J, Allen N, Beral V, Burton P, Danesh J, Downey P, Elliott P, Green J, Landray M, Liu B, Matthews P, Ong G, Pell J, Silman A, Young A, Sprosen T, Peakman T, Collins R (2015). UK biobank: an open access resource for identifying the causes of a wide range of complex diseases of middle and old age. PLoS medicine..

[31] UK Chief Medical Officers. UK chief medical officers’ low risk drinking guidelines. https://assets.publishing.service.gov.uk/government/uploads/system/uploads/attachment_data/file/545937/UK_CMOs__report.pdf; 2016.

[32] World Health Organization, Food and Agriculture Organization of the United Nations. Diet, nutrition and the prevention of chronic diseases. https://www.who.int/nutrition/publications/obesity/WHO_TRS_916/en/; 2003.

[33] Wang DD, Li Y, Bhupathiraju SN, Rosner BA, Sun Q, Giovannucci EL, Rimm EB, Manson JAE, Willett WC, Stampfer MJ, Hu FB (2021). Fruit and vegetable intake and mortality: results from 2 prospective cohort studies of US men and women and a meta-analysis of 26 cohort studies. Circulation..

[34] World Health Organisation. Physical activity 2020 [Available from: https://www.who.int/news-room/fact-sheets/detail/physical-activity.

[35] Lu Y, Hajifathalian K, Ezzati M, Woodward M, Rimm EB, Global Burden of Metabolic Risk Factors for Chronic Diseases C (2014). Metabolic mediators of the effects of body-mass index, overweight, and obesity on coronary heart disease and stroke: a pooled analysis of 97 prospective cohorts with 1.8 million participants. Lancet.

[36] Weir CB, Jan A (2020). BMI classification percentile and cut off points.

[37] Plummer M (2004). Improved estimates of floating absolute risk. Statistics in Medicine..

