# Supplementary material for: Obesity, metabolic risk and adherence to healthy lifestyle behaviours: prospective cohort study in the UK Biobank
Source: BMC Med. 2022 Feb 15;20:65. doi: 10.1186/s12916-022-02236-0 (PMC8845299; doi:10.1186/s12916-022-02236-0)
Supplement: Supplementary file 1 — Additional file 1: Figure S1. Flow chart through the study. [file 12916_2022_2236_MOESM1_ESM.docx]

Figure S1: Flow chart through the study

502,505 original participants recruited between 2006 to 2010

339,902 included in analysis

- 17,376 died
  - 4,653 fatal CVD
- 29,517 Incident CVD

Exclusion Criteria

- BMI <18.5kg/m^2^ or missing (n=5,146)
- Prior CVD (n=37,108)
- Pregnant (n=368)
- Smoking status missing (n=2,211)
- Alcohol intake missing (n=101,397)
- Fruit and vegetable intake missing (n=5,852)
- Physical activity missing (n=10,521)
