# Supplementary material for: Obesity, metabolic risk and adherence to healthy lifestyle behaviours: prospective cohort study in the UK Biobank
Source: BMC Med. 2022 Feb 15;20:65. doi: 10.1186/s12916-022-02236-0 (PMC8845299; doi:10.1186/s12916-022-02236-0)
Supplement: Supplementary file 2 — Additional file 2: Table S1. Complete Data vs Missing Data Participant Characteristics at Baseline. [file 12916_2022_2236_MOESM2_ESM.docx]

Table S1: Complete Data vs Missing Data Participant Characteristics at Baseline

|  |  |  |
| --- | --- | --- |
| Factor | Complete Data | Missing Data |
| N | 339902 | 119981 |
| Any death* | 17387 (5.1%) | 8121 (6.8%) |
| Incident cardiovascular disease (CVD) | 29545 (8.7%) | 12922 (10.8%) |
| Male | 161849 (47.6%) | 41478 (34.6%) |
| Age, mean (SD) | 56.2 (8.0) | 56.2 (8.2) |
| Body mass index (BMI), mean (SD) | 27.1 (4.4) | 28.1 (5.4) |
| Higher education | 144035 (42.4%) | 38751 (32.3%) |
| White ethnicity | 326745 (96.1%) | 106738 (89.0%) |
| Current smoking | 33703 (9.9%) | 13455 (11.2%) |
| Low fruit and vegetable intake | 238184 (70.1%) | 72003 (60.0%) |
| High alcohol consumption | 188151 (55.4%) | 8742 (7.3%) |
| Low physical activity | 130360 (38.4%) | 39169 (32.6%) |
| Hypertension | 173489 (51.0%) | 60992 (50.8%) |
| Diabetes | 12434 (3.7%) | 7859 (6.6%) |
| Hyperlipidaemia | 138065 (40.6%) | 50,178 (41.8%) |
| Post menopause | 106899 (31.4%) | 47206 (39.3%) |
| Family history of diabetes | 60259 (17.7%) | 25733 (21.4%) |
| Family history of CVD | 200113 (58.9%) | 69550 (58.0%) |

**Figures are n(%) unless otherwise state
