# Supplementary material for: Obesity, metabolic risk and adherence to healthy lifestyle behaviours: prospective cohort study in the UK Biobank
Source: BMC Med. 2022 Feb 15;20:65. doi: 10.1186/s12916-022-02236-0 (PMC8845299; doi:10.1186/s12916-022-02236-0)
Supplement: Supplementary file 3 — Additional file 3: Table S1. Cox Proportional Hazards Models for Risk of All-Cause Mortality, Cardiovascular Mortality, or Cardiovascular Disease Diagnosis by Adherence to 0, 1, 2, 3, or 4 Healthy Lifestyle Behaviours Excluding First 2 Years of Follow Up. Table S2. Cox Proportional Hazards Models for Risk of All-Cause Mortality, Cardiovascular Mortality, or Cardiovascular Disease Diagnosis by Adherence to 0, 1, 2, 3, or 4 Healthy Lifestyle Behaviours, Stratified by BMI Group, Excluding First 2 Years of Follow Up. Table S3. Cox Proportional Hazards Models for Risk of All-Cause Mortality, Cardiovascular Mortality, or Cardiovascular Disease Diagnosis by According to Presence or Absence of Metabolic Risk Factors, Stratified by BMI Group, Excluding First 2 Years of Follow Up. [file 12916_2022_2236_MOESM3_ESM.docx]

Table S1: Cox Proportional Hazards Models for Risk of All-Cause Mortality, Cardiovascular Mortality, or Cardiovascular Disease Diagnosis by Adherence to 0, 1, 2, 3, or 4 Healthy Lifestyle Behaviours Excluding First 2 Years of Follow Up

|  | | Total All-Cause Mortality | | Cardiovascular Mortality | | Cardiovascular Disease | |
| --- | --- | --- | --- | --- | --- | --- | --- |
| Number of healthy behaviours | N | Cases | HR (95% CI)* | Cases | HR (95% CI) | Cases | HR (95% CI) |
| 0 | 29925 | 2196 | 1.83 (1.76-1.91) | 700 | 1.86 (1.73-2.01) | 3048 | 1.39 (1.34-1.44) |
| 1 | 92628 | 5203 | 1.45 (1.41-1.49) | 1550 | 1.43 (1.36-1.50) | 7996 | 1.22 (1.20-1.25) |
| 2 | 117851 | 5102 | 1.20 (1.17-1.24) | 1437 | 1.17 (1.12-1.24) | 8731 | 1.15 (1.13-1.17) |
| 3 | 77098 | 2906 | 1.08 (1.04-1.12) | 777 | 1.07 (0.99-1.15) | 5059 | 1.09 (1.06-1.12) |
| 4 (reference) | 21155 | 735 | 1.00 (0.93-1.08) | 191 | 1.00 (0.87-1.15) | 1253 | 1.00 (0.95-1.06) |

*HR = Hazard Ratio CI = Confidence Interval (floating)

Multivariable Hazard Ratio adjusted for age, sex, ethnicity, Townsend deprivation score, education, region, family history of cardiovascular disease, family history of diabetes, menopausal status, BMI group

Table S2: Cox Proportional Hazards Models for Risk of All-Cause Mortality, Cardiovascular Mortality, or Cardiovascular Disease Diagnosis by Adherence to 0, 1, 2, 3, or 4 Healthy Lifestyle Behaviours, Stratified by BMI Group, Excluding First 2 Years of Follow Up

|  | | All-Cause Mortality | | Cardiovascular Mortality | | Cardiovascular Disease | |
| --- | --- | --- | --- | --- | --- | --- | --- |
| Number of healthy behaviours | N | Cases | HR (95% CI)* | Cases | HR (95% CI) | Cases | (95% CI) |
| *BMI 18.5-24.9 kg/m^2^* |  |  |  |  |  |  |  |
| 0 | 8121 | 605 | 2.51 (2.32-2.72) | 150 | 2.52 (2.15-2.96) | 619 | 1.56 (1.44-1.68) |
| 1 | 28719 | 1428 | 1.70 (1.62-1.79) | 330 | 1.63 (1.46-1.82) | 1717 | 1.23 (1.17-1.29) |
| 2 | 41892 | 1486 | 1.29 (1.22-1.36) | 329 | 1.23 (1.10-1.37) | 2197 | 1.14 (1.10-1.19) |
| 3 | 30516 | 957 | 1.15 (1.08-1.23) | 200 | 1.07 (0.93-1.23) | 1402 | 1.03 (0.97-1.08) |
| 4 (reference) | 8866 | 246 | 1.00 (0.88-1.13) | 54 | 1.00 (0.76-1.31) | 398 | 1.00 (0.91-1.10) |
| *BMI 25-29.9 kg/m^2^* |  |  |  |  |  |  |  |
| 0 | 13887 | 9145 | 1.88 (1.76-2.00) | 275 | 2.06 (1.83-2.32) | 1382 | 1.64 (1.55-1.73) |
| 1 | 42580 | 2335 | 1.54 (1.47-1.60) | 647 | 1.63 (1.51-1.76) | 3792 | 1.48 (1.43-1.53) |
| 2 | 51381 | 2264 | 1.32 (1.27-1.38) | 567 | 1.31 (1.20-1.42) | 3965 | 1.37 (1.33-1.42) |
| 3 | 31911 | 1298 | 1.26 (1.19-1.33) | 329 | 1.32 (1.18-1.47) | 2293 | 1.35 (1.29-1.40) |
| 4 | 8425 | 297 | 1.09 (0.98-1.23) | 83 | 1.31 (1.06-1.63) | 539 | 1.22 (1.12-1.33) |
| *BMI 30-34.9 kg/m^2^* |  |  |  |  |  |  |  |
| 0 | 6078 | 475 | 2.16 (1.97-2.36) | 164 | 2.79 (2.40-3.26) | 770 | 2.10 (1.96-2.26) |
| 1 | 16190 | 1066 | 1.87 (1.76-1.99) | 331 | 2.22 (2.00-2.48) | 1816 | 1.92 (1.83-2.01) |
| 2 | 18435 | 979 | 1.59 (1.49-1.69) | 308 | 1.97 (1.76-2.20) | 1855 | 1.84 (1.75-1.92) |
| 3 | 10915 | 463 | 1.32 (1.21-1.45) | 138 | 1.65 (1.39-1.95) | 972 | 1.72 (1.61-1.83) |
| 4 | 2842 | 127 | 1.42 (1.19-1.69) | 28 | 1.36 (0.94-1.97) | 230 | 1.61 (1.41-1.83) |
| *BMI*≥*35 kg/m^2^* |  |  |  |  |  |  |  |
| 0 | 1839 | 171 | 2.82 (2.43-3.28) | 64 | 4.06 (3.18-5.19) | 277 | 2.84 (2.53-3.20) |
| 1 | 5139 | 374 | 2.34 (2.12-2.59) | 139 | 3.46 (2.93-4.09) | 671 | 2.60 (2.41-2.80) |
| 2 | 6143 | 373 | 2.10 (1.90-2.32) | 137 | 3.25 (2.75-3.85) | 714 | 2.54 (2.36-2.73) |
| 3 | 3711 | 188 | 1.79 (1.55-2.06) | 69 | 2.94 (2.32-3.72) | 392 | 2.43 (2.20-2.68) |
| 4 | 1022 | 65 | 2.24 (1.75-2.85) | 21 | 3.27 (2.13-5.02) | 86 | 1.89 (1.53-2.34) |

*HR = Hazard Ratio CI = Confidence Interval (floating)

Multivariable Hazard Ratio adjusted for age, sex, ethnicity, Townsend deprivation score, education, region, family history of cardiovascular disease, family history of diabetes, menopausal status

Table S3: Cox Proportional Hazards Models for Risk of All-Cause Mortality, Cardiovascular Mortality, or Cardiovascular Disease Diagnosis by According to Presence or Absence of Metabolic Risk Factors, Stratified by BMI Group, Excluding First 2 Years of Follow Up

|  | | All-Cause Mortality | | Cardiovascular Mortality | | Cardiovascular Disease | |
| --- | --- | --- | --- | --- | --- | --- | --- |
| BMI kg/m^2^ | N | Cases | HR (95% CI)* | Cases | HR (95% CI) | Cases | HR (95% CI) |
| *18.5-24.9* |  |  |  |  |  |  |  |
| Metabolically “Healthy”**  (Reference) | 56281 | 1546 | 1.00 (0.95-1.05) | 250 | 1.00 (0.88-1.13) | 1707 | 1.00 (0.95-1.05) |
| Metabolically “Unhealthy” | 61873 | 3176 | 1.10 (1.06-1.14) | 813 | 1.56 (1.46-1.68) | 4626 | 1.54 (1.50-1.59) |
| *25-29.9* |  |  |  |  |  |  |  |
| Metabolically “Healthy” | 42816 | 1347 | 1.00 (0.95-1.05) | 239 | 1.01 (0.89-1.15) | 1795 | 1.18 (1.12-1.23) |
| Metabolically “Unhealthy” | 105368 | 5792 | 1.06 (1.03-1.08) | 1662 | 1.59 (1.51-1.66) | 10176 | 1.78 (1.75-1.82) |
| *30-34.9* |  |  |  |  |  |  |  |
| Metabolically “Healthy” | 10494 | 343 | 1.07 (0.96-1.19) | 78 | 1.40 (1.12-1.75) | 515 | 1.45 (1.33-1.58) |
| Metabolically “Unhealthy” | 43966 | 2767 | 1.27 (1.22-1.32) | 891 | 2.14 (2.01-2.29) | 5128 | 2.28 (2.22-2.35) |
| *≥35* |  |  |  |  |  |  |  |
| Metabolically “Healthy” | 2646 | 104 | 1.55 (1.28-1.89) | 23 | 2.15 (1.43-3.24) | 151 | 2.07 (1.76-2.43) |
| Metabolically “Unhealthy” | 15208 | 1067 | 1.64 (1.54-1.74) | 407 | 3.46 (3.13-3.81) | 1989 | 3.04 (2.91-3.18) |

*HR = Hazard Ratio CI = Confidence Intervals (floating)

**Metabolically “healthy” includes patients without a diagnosis of hypertension, diabetes, or high cholesterol. Metabolically “unhealthy” includes patients with a diagnosis of hypertension and/or diabetes and/or high cholesterol

Multivariable Hazard Ratio adjusted for age, sex, ethnicity, Townsend deprivation score, education, region, family history of cardiovascular disease, family history of diabetes, menopausal status
