# Supplementary material for: Obesity, metabolic risk and adherence to healthy lifestyle behaviours: prospective cohort study in the UK Biobank
Source: BMC Med. 2022 Feb 15;20:65. doi: 10.1186/s12916-022-02236-0 (PMC8845299; doi:10.1186/s12916-022-02236-0)
Supplement: Supplementary file 4 — Additional file 4:. Statistical Analysis Plan: Healthy behaviours, cardiovascular disease and all-cause mortality in the UK Biobank. [file 12916_2022_2236_MOESM4_ESM.docx]

# **Statistical Analysis Plan: Healthy behaviours, cardiovascular disease and all-cause mortality in the UK Biobank**

## **Aims**

To investigate the association between adherence to healthy behaviours and the risk of total cardiovascular disease (CVD), fatal CVD and total mortality by BMI group in the UK Biobank.

## **Outcomes**

The main outcomes are incidence of any form of CVD, fatal CVD, and total mortality. Incident cardiovascular disease will be defined as a hospital admission or death with ICD-10 (international classification of diseases, 10th revision) codes including: coronary heart disease (CHD; I20-I25, K49, K50, K75, K40-K46), congestive heart failure or cardiomyopathy (CHF; I50, I50.1, 150.9, I11.0, I13.0, I13.2, I42, I43.1), and total stroke (I60-I64). For total fatal CVD events, we will use I00-I25, I27-I88, and I95-I99. Total mortalities will be obtained from death registries.

## **Exposures**

Four healthy behaviours will be classed as exposures all measured at cohort inception from a questionnaire administered then. These will be analysed separately and then as a total number of healthy behaviours. No data to estimate change in these variables is available:

1. *Smoking status.* This will be classified as “Never”, “Current” and “Previous” as per smoking status self-reported by the UK Biobank participants. Being a never smoker is defined as a healthy behaviour.
2. *Alcohol intake frequency*. To be divided into “None”, “Occasional” (<1 unit/ week), “Moderate” (1-14 units/ week) and “Heavy” (>14 units/ week). A healthy behaviour for this study is defined as ‘none’ or ‘occasional’ alcohol intake. Data was calculated from participants’ responses to estimate consumption of red wine^†^; champagne and white wine ^†^; beer and cider^†^; spirits^†^; fortified wine^†^.
3. *Fruit and Vegetable Score.* The sum of the following variables was calculated: Fresh fruit intake^†^, raw vegetable intake^†^, cooked vegetable intake. These were summed and a healthy behaviour defined as 5 or more servings of fruit and vegetables a day.
4. *Physical Activity.* Derived MET score from number of days/week of vigorous physical activity 10+ minutes^†^; Duration of vigorous activity; number of days/week of moderate physical activity 10+ minutes^†^; duration of moderate activity and number of days/week walked 10+ minutes^†^; duration of walks ^†.^ A healthy behaviour was defined as 150 or more minutes of moderate activity, or 75 minutes or more of vigorous activity a week, or a combination of the two, as defined by the UK CMO physical activity guidelines.

**BMI**

Participants will be stratified into four BMI categories as follows; BMI 18.5 -24.9, 25-29.9, 30-34.9 and >=35 kg/m^2^. The category 18.5-24.9 kg/m^2^will be the reference group.

## **Exclusions /Inclusions**

List of exclusions:

- Pregnancy
- BMI <18.5 kg/m^2^.
- Participants who have a diagnosis of CVD at baseline. This includes the outcome variables of coronary heart disease, congestive heart failure, cardiomyopathy and stroke.

## **Covariates & Effect Modifiers**

### 1. Specific coding of covariates

Population demographics:

- Age: time underlying time co-variate in the Cox model.
- Ethnicity: White, Black, Asian, Other
- Townsend score (quintiles 1-5, with lower score representing greater affluence)
- Education: categorical (1: higher degree, 2: any school degree, 3: vocational qualifications, 0: none of the above)
- Sex (Male:0; Female: 1)
- Region of UK

Health factors:

- Family history of diabetes (No:0; Yes: 1)
- Family history of CVD (No:0; Yes: 1)
- Menopausal status (men:0, premenopausal women: 1, postmenopausal women:2)

## 2. Plan for dealing with missing data

- Participants with missing data in key variables will be dropped:
  - If the exposure measurements smoking status, alcohol intake, fruit and vegetable score, physical activity score were missing or unknown.
  - Other key confounders in the model: Townsend score, education, age, sex, region of UK, family history of diabetes or hypertension, if missing, we will create a category called “Unknown”
  - If menopausal status is missing, age >=55 will be used as a proxy.
  - If key stratification variable BMI was missing or unknown.

## **Planned main analyses**

## 1. *Descriptive statistics*

For continuous variables: mean, standard deviation

For categorical variables: 2 x k tables, frequency distributions

## *2. Multivariable analysis*

*PRIMARY ANALYSIS*: to investigate the association between number of healthy behaviours and the total CVD events, fatal CVD and total mortality overall using sex and region stratified models

### *SECONDARY ANALYSIS:* to investigate the association between number of healthy behaviours and the risk of total CVD events, fatal CVD and total mortality in the 4 BMI strata using sex and region stratified models

For both the primary and secondary analysis the number of healthy behaviours (0,1,2,3 or 4) out of a total of 4 will be used as the main exposure, using 4 as the reference category

### For both the primary and secondary analysis:

1. Timescale for the Cox model:

Time from inception to incident of disease will be represented by age. We will use the following command in Stata before the model: stset age_out, id(ID) fail(outcome) enter(age_in)

1. Adjustment:

We will use a multivariable confounding model adjusted for: Age, Townsend score, education, sex, region of UK, family history of diabetes or hypertension and menopausal status.

## **Sensitivity analyses**

- Removing the first 2 years of follow up to try to limit the potential for reverse causality

## **Supplementary analyses**

Some research has suggested that it is possible to have obesity and be ‘metabolically healthy’. In this study ‘metabolically healthy’ will be defined as those participants without a diagnosis of hypertension, diabetes or high cholesterol. We will explore the following questions:

1. How prevalent is ‘metabolically healthy’ status amongst participants who have obesity?
   - We will determine the % of the population with BMI>30 with and without metabolic complications
2. Does ‘metabolically healthy’ obesity lower the incidence of total CVD, fatal CVD and total mortality compared to ‘non-metabolically healthy’ obesity? How does the incidence of total CVD, fatal CVD and total mortality in ‘metabolically healthy’ obesity category compare to the incidence of total CVD, fatal CVD and total mortality in the ‘normal’ BMI category?
   - We will re-run our models (Secondary analyses) to have an interaction between BMI with presence/absence of metabolic complications

**In summary:**

*Primary analysis*

1. CVD = #beh + BMI + cova

*Secondary analysis*

1. CVD= #behaviours*BMI group + covariates
2. CVD= individual behaviours*BMI group + covariates

*Supplementary analysis*

1. CVD=#behaviours*BMI group*Metabolic Conditions + covariates
